# Biocomputation: Moving Beyond Turing with Living Cellular Computers

**DOI:** 10.1145/3635470

**Published:** 2024-05-23

**Authors:** Ángel Goñi-Moreno

**Affiliations:** Centro de Biotecnología y Genómica de Plantas, Universidad Politécnica de Madrid (UPM)- Instituto Nacional de Investigación y Tecnología Agraria y Alimentaria (INIA/CSIC), Madrid, Spain

**Keywords:** Biocomputation, cellular computing, unconventional computing, synthetic biology, systems biology, computing models.

## Abstract

The design and implementation of new-to-nature living systems with human-defined computing capabilities is now a routine process. Cells, such as bacteria, can be rationally modified to respond to a set of inputs and deliver outputs based on algorithmic rules that have been synthetically encoded into their genomes; and the resulting cellular computers are used in various domains, ranging from medical to environmental. With the era of synthetic biology, the last 20 years have seen tremendous advancements in molecular biology and microbiology, allowing us to engineer living systems with unprecedented precision. But while combinatorial functions are currently the main focus of programming living cells, biological systems have so much more to offer. It is now the time to explore, characterize, and exploit the full computing power of living systems. How do they handle stateful computations? What classes of problems can they solve? What is the complexity of gene regulation and evolution as problem-solving processes? These (non-trivial) questions, and many more, demand more attention from the computer science community. This article advocates for leveraging the synergies between theoretical computer science and synthetic biology to create more powerful cellular computers and move beyond conventional Turing computation. The limits of what can be computed with synthetic biological systems are still being explored.

## 1 Background and motivation

It is a well-known story that theoretical computer science and biology have been drawing inspiration from each other for decades. While computer science has tried to mimic the functioning of living systems to develop computing models, including automata, artificial neural networks, and evolutionary algorithms, biology has used computing as a metaphor to explain the functioning of living systems^4^. For example, biologists have used Boolean logic to conceptualize gene regulation since early 70s, when Jacques Monod wrote the inspirational statement “…like the workings of computers”^40^.

**Figure 1:**
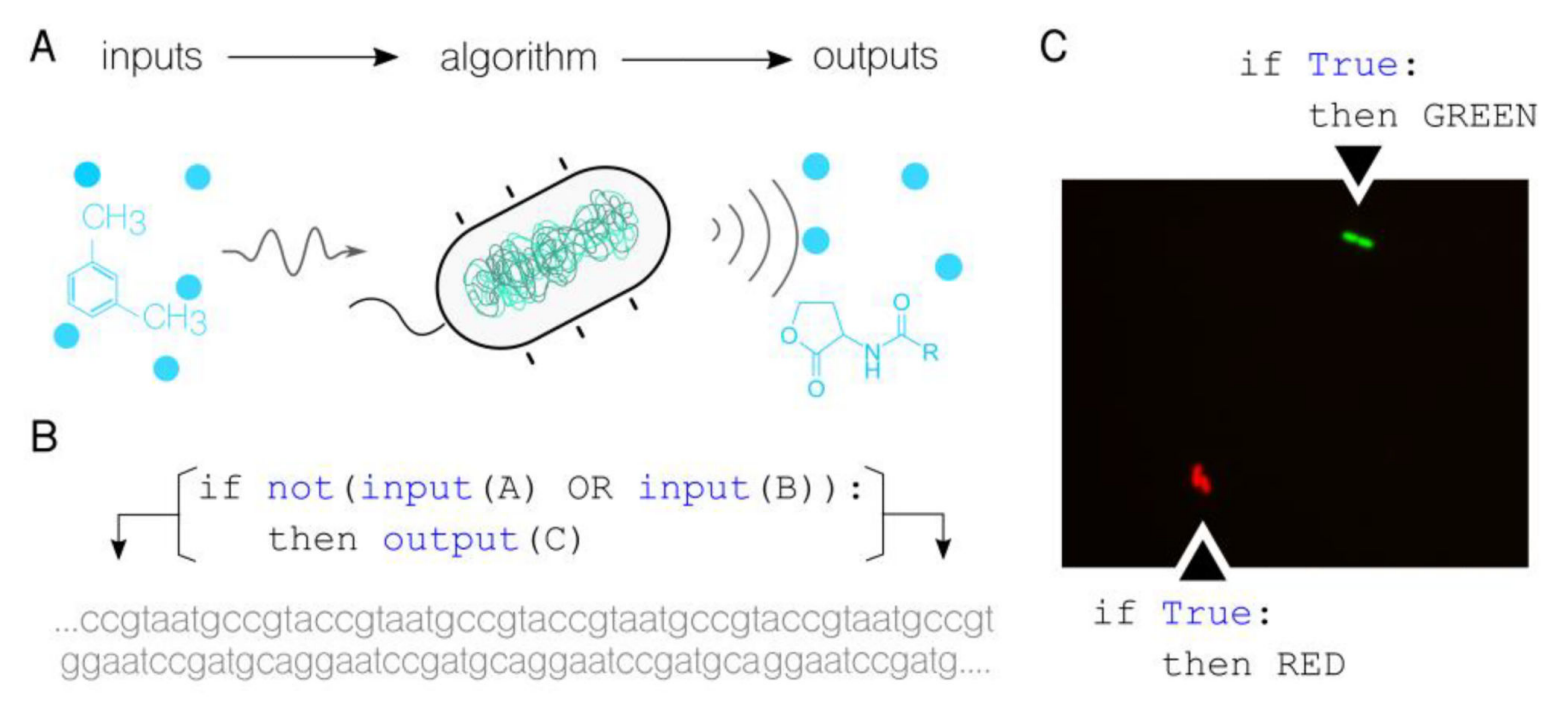
Programming bacteria to perform living Boolean logic functions. **A**. Cells possess the ability to sense a wide range of physicochemical inputs and deliver outputs according to algorithmic rules encoded in their DNA. This input-algorithm-output relationship is crucial to the concept of computation. **B**. Combinatorial Boolean logic functions can be integrated into a cell's genome. For instance, being A and B two chemical input signals, and C the expression product of a given output gene, the function if ¬ (A ∨ B) then C can be encoded into a DNA sequence, which can then be introduced into a living cell. **C**. In the example shown, simple logic statements are implemented in the bacterium *Pseudomonas putida* and visualized using fluorescence microscopy. The cells at the top have been programmed to express a green fluorescent protein irrespective of the input, while the cells at the bottom have been programmed to produce red fluorescent proteins as their output.

The standpoint of this article is that information processing is the link between computer science and molecular biology. Information and its processing lie at the core of both fields. In computer science, a model of computation such as finite state machines or Turing machines defines how to generate output from a set of inputs and a set of rules or instructions. Similarly, biological systems (like the bacterial cell in Figure 1A) sense and react to input stimuli to generate a response according to their internal configuration. Using synthetic biology^6^, it is now possible to modify the specific nature of each of these steps in biological systems (e.g., edit the DNA of living cells to sense new inputs) allowing for the programming of information processing devices with living matter^9^. This exciting breakthrough not only provides possibilities for applications that traditional computers cannot reach, but it also challenges the traditional idea of computation and what can be computed. This fascinating concept has the potential to take computer science to new frontiers, paving the way for future advances and discoveries.

Programming living systems is fundamentally different from programming non-living systems due to the unique physical substrate that is used. Unlike conventional technology that has been designed and built by humans, living systems have evolved through natural selection. This means that the tools and techniques we use to program conventional technology are predictable and known, with clear limits to what can be achieved. However, living matter, such as cells, genes, and molecules, have not been artificially built by us and the rules that govern their functioning are not fully understood. While we can build simple models of computation, like Boolean logic^52^, using genetic networks, more complex computations still pose a challenge. To develop more advanced biocomputing, we need to continue expanding our knowledge of how molecular systems work and develop new theories of computation that are better suited for living systems and go beyond conventional Turing computing.

Recent advances in molecular biology have led to significant developments in biocomputing, with four key stages identified in the field's progress. Initially, during the early 90s, theoretical advancements and conceptual ideas dominated the field^17^ as the technology needed for experimentation wasn't yet in place. This was followed by a period of progress, until the late 90s, where both molecular biology and biocomputing advanced significantly, leading to the first experiments on DNA computing. In 1994, Leonard Adleman performed an iconic experiment by using molecular operations and DNA strands to solve an instance of the HPP problem^3^. The third stage occurred in the late 90s and early 2000s with the advent of synthetic biology, which allowed the implementation of logic gates, switches, and feedback loops in bacteria^1, 23, 32^. The fourth stage, from the late 2000s onwards, has seen significant technical progress in cloning and DNA synthesis, which enables the creation of precise genetic constructs^39^. Notably, theoretical developments now lag behind experimental capabilities, a scenario opposite to that before the 90s. This reinforces the core message of this article: the intersection of theoretical computer science and (synthetic) biology has a unique opportunity to unlock new possibilities

In a recent publication we coined the term *cellular supremacy*^29^ to refer to the implementation of cellular computers that could outperform classical computers at specific tasks—a parallel concept to the quantum supremacy. These tasks are unlikely to fall within the domains where silicon-based computers excel, particularly in efficiently solving mathematical problems. However, there exist domains where classical computers are virtually useless. For example, consider a decision problem like assessing the danger posed to a plant ecosystem by the combination of two environmental pollutants, x and y. In such cases, a cellular computer residing within that ecosystem could offer a solution and, more importantly, suggest remedial actions for the plant if the answer is affirmative. Such a computing device has the added advantage of adapting to environmental conditions and reproducing itself into a larger number of similar functions.

Similar examples can be readily identified in other domains, such as addressing medical issues.

In what follows, this article breaks down the basic concepts for building cellular computers and suggests exciting new ways of improvement.

## 1 Programming basic Boolean functions

Since the emergence of synthetic biology, implementing Boolean logic functions has become a major focus for biocomputing. This makes sense because a single gene can either be active (i.e., *expressed*) or not at any given time, allowing us to abstract two values: on/off or 1/0. Additionally, from an experimental molecular biology standpoint, this process is relatively easy, and the technology is readily available^41^. So, what are the basics for implementing novel, living combinatorial instructions?

The definition of information in living systems is a subject of ongoing debate^2, 44^, and for the sake of simplicity, let's assume that non-active information is stored in DNA as genes. In other words, the functions required for the proper functioning of the cell are encoded into genetic sequences, but these functions remain inactive until they are *expressed*. It's important to note that this concept of biological information—information that is useful to a biological system—differs from the definition of information in the field of DNA data storage^16^. In DNA data storage, the aim is to encode non-biological information (information not useful to any biological system) into genetic sequences, utilizing DNA as an alternative storage device. In this discussion, we are specifically referring to the former definition.

When a gene is expressed, it produces a protein that performs the function encoded in that information. In other words, the gene won't perform its function unless it's expressed. Gene expression follows a Boolean approach, and genes can be turned on and off as needed. For example, a gene encoding function C can be activated only when specific inputs A and B are combined, as shown in Figure 1B. All variables in this process can be modified to a great extent: inputs, outputs, and the algorithmic rules that process them can all be defined.

Figure 1C showcases the simplest logic program ever implemented in cells of the soil bacteria Pseudomonas putida^22^. The image, captured using fluorescence microscopy, shows growing cells - two single cells at the start of the experiment that eventually divided into four by the time the picture was taken. The cells at the top emit a bright green signal due to the green fluorescent proteins they're expressing, while the cells at the bottom produce red fluorescent proteins. Both sets of cells follow the same logic program, which states that the output should be expressed regardless of the input (in this case, no input is defined, making it a True statement). It is important to note that this program, which is encoded into a DNA sequence, isn't found in these wild organisms naturally. Instead, it has been artificially added to their genome.

Since the late 1990s, researchers have been implementing genetic NOT logic functions and other Boolean logic gates in bacteria^54^, yeasts^15^, and mammalian cells^7^. Over the years, these implementations have been refined in various ways. For instance, the gates were made more modular so they could be linked together to build a larger genetic circuit. They also became more standardized and better characterized, making the design process more automated. Additionally, scientists explored various cellular machinery to expand the toolkit used to construct these gates, including invertases, post-transcription reactions, and even populations of cells^26, 46^ rather than single ones. It's interesting to compare the computing power of cellular computers with the potential of living systems. A fairly large but reasonable logic function that can be implemented in a cell may require 6 to 8 genes, and an *Escherichia coli* cell has more than 4000 genes. This suggests that we have only just begun to unlock the computing power of living systems. Despite this, designing and implementing Boolean logic functions in living cells is a routine process today.

However, combinatorial logic is a limited model of computation when compared to finite state machines or Turing machines. And living systems have much more to offer in terms of information processing. So, what other models have been implemented? And

## 2 Living models of computation

Models of computation are theoretical concepts that help us understand what problems can be solved and how, under specific conditions. They were crucial in the development of modern silicon-based computers powered by electricity. However, in biocomputing, we need to approach things differently: living organisms evolved their own algorithms, and we need to figure out how they process information in ways that surely differ from human-made machines. This challenge goes from the comparison of genetic networks to electronic circuits and extends to the comparison of brain processes to microprocessors^31^.

Despite our incomplete knowledge, we have a wide range of biological language primitives—a software term that refers to simple functional elements—at our disposal to build computing devices. While implementing more complex models of computation than simple Boolean logic in living cells is rare, researchers have made progress in this field. One promising approach is building state machines, devices that can transition between a finite number of states in response to specific inputs.

**Figure 2:**
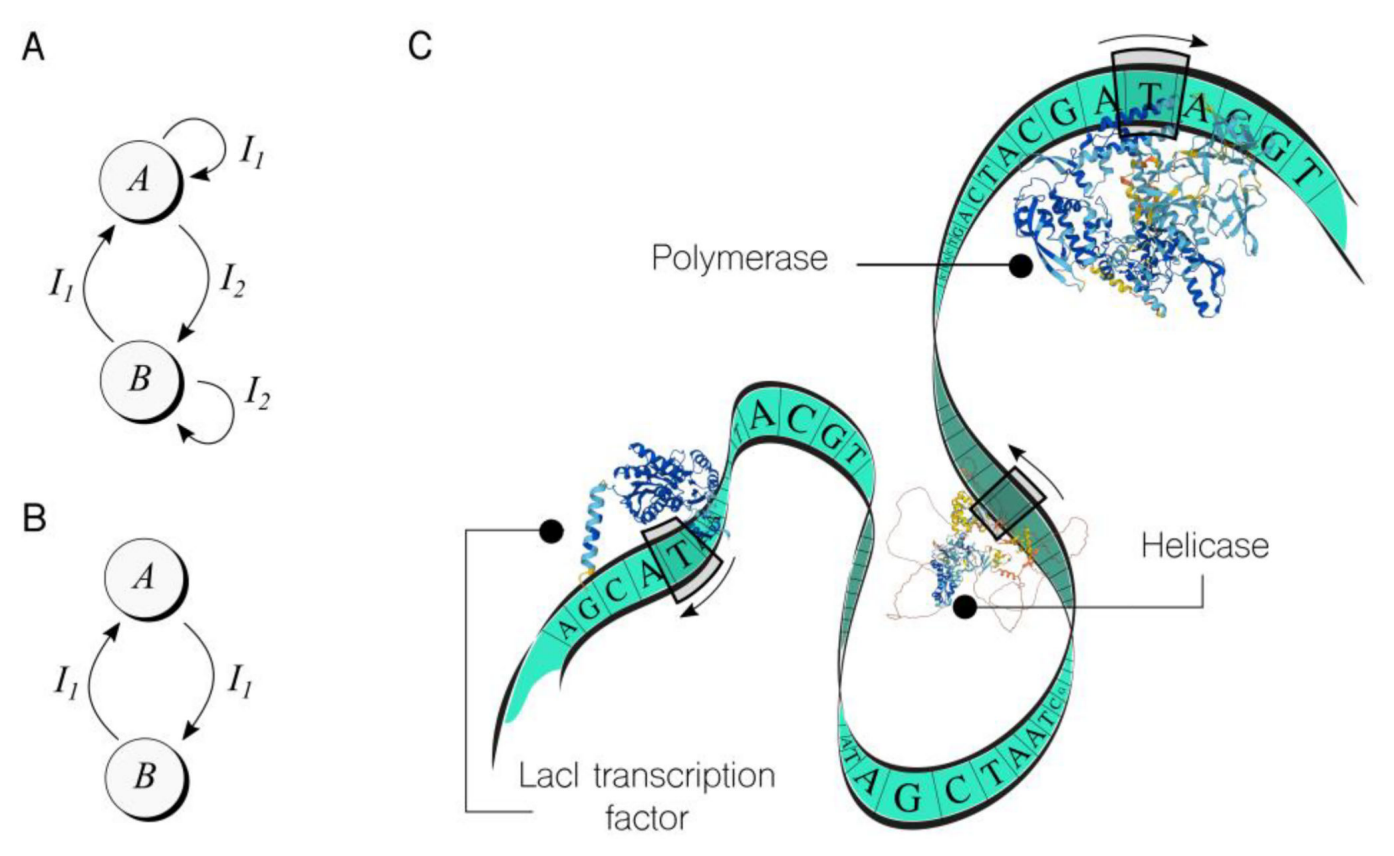
Implementing models of computation in cellular computers beyond combinatorial logic. **A**. Move beyond simple Boolean logic and explore powerful models of computation like Finite State Machines. Genetic toggle switches, for example, use two chemical inputs (*I*_x_) to toggle between stable states A and B, as shown in the sketch. **B**. Genetic switches that use only one input to change states are more challenging to implement, but there are already examples. **C**. Imagine a DNA sequence as a tape-like data storage that can be read and processed by enzymes in a Turing-like system of computation. Although this system is not yet fully understood, nor formalized as a computing device, it offers exciting possibilities for unlocking the true information-processing power of living systems. A noteworthy example in this context is the four-decade-old conceptualization of such an *enzymatic apparatus* as a computing system capable of executing logically reversible functions^10^—an approach that has recently undergone experimental analysis.^50^ [Protein structures generated with AlphaFold https://alphafold.ebi.ac.uk] what models are yet to be explored?

One of the earliest examples of such a machine was the genetic toggle switch^23^, engineered in 2000 in the bacterium *Escherichia coli*. This switch had two stable states, labelled *A* and *B*, as shown in Figure 2A, and two inputs, *I*_*1*_ and *I*_*2*_, that toggled the genetic device between states. Each state was implemented using a different expression system (i.e., a gene and other required sequences), and the inputs were small molecules that could be externally added on demand. Since then, there have been many versions and optimizations of the toggle switch, as well as theoretical studies on how to improve its performance^42^.

Another noteworthy example is the push-on push-off switch, shown in Figure 2B, implemented also into *Escherichia coli* cells^34^. This state machine requires only one input signal to transition between states, although it increases the complexity of the implementation by adding extra control signals. As a result, cells performed the switching in response to one external stimuli.

More adventurous biocomputing efforts move beyond traditional models and implement neuro-inspired architectures using genetic material^33^, creating biological versions of artificial neural networks and perceptrons^43^ capable of solving problems using fuzzy logic and reversibility. While these approaches have been successful, and possibly closer to natural algorithms, there are likely many other living models of computation yet to be discovered. In fact, some researchers have proposed the idea of moving beyond the limitations of Turing machines by utilizing biological processes^36^, although practical implementations of such systems have yet to emerge.

Imagine DNA as a Turing Machine (TM). In this analogy, DNA is like the infinite tape in a TM with symbols written in it. However, in the case of DNA, the tape is limited to four symbols: A, C, G, and T. There are many heads (enzymes) sliding along the tape in both directions, reading, writing, and performing other tasks – mimicking a multi-head TM. These heads work independently and asynchronously, and their behaviour depends on specific rules or instructions that are unique to each head. Moreover, they do not just slide along the DNA, but also detach from it to diffuse through the volume of the cell, then bind again to the tape and continue sliding.

In Figure 2C, a cartoon depicts a DNA sequence as a Turing Machine. The figure shows three specific types of heads—from the many available in living systems. For example, polymerases are enzymes that read the DNA until they identify a specific string, then generate an RNA molecule that corresponds to the following symbols until a stop string is encountered. This process is called transcription. Helicases are another type of head that recognizes a string called the origin of replication and then helps recruit other cellular machinery to replicate the DNA, leading to two tapes in our TM analogy. The third type of head shown in Figure 2C is a LacI Transcription Factor (TF). TFs are a key part of the synthetic biology toolkit^27^, as they are used to trigger or block the action of synthetic genes and propagate the on/off motif through a cascade of functional genetic devices.

One interesting aspect of this bio-TM is that most of its elements can be programmed. For example, the symbols on the tape can be edited, and the presence or absence of TFs can be modulated – even non-digital stochastic amounts, leading to analogue^19^ and neuromorphic^47^ computing approaches. The rules that govern various heads, such as the polymerases, can also be edited. For instance, polymerases can be made dependent on a specific combination of inputs, which includes this head within the computation already.

While the concept of DNA as a TM is definitely intriguing, it is still an oversimplification of the complexity of biological systems. Nevertheless, this analogy provides a useful framework for understanding the computational abilities of living organisms and how to engineer them to perform specific tasks. For example, by studying the biological machinery of living systems, we can discover new models of computation that can be used to build cellular computers that may perform better than current silicon-based computers—towards the cellular supremacy^29^.

Up until now, this article has focused on computer science and its analysis of information from a software perspective. However, cellular computing is not only concerned with software, but also with the physical implementation of modified organisms, which involves hardware issues—and, therefore, the field of engineering^5^.

## 3 Hardware, software or none

The debate on whether hardware or software provides the better conceptual framework for formalizing biological systems' performance is ongoing^18^. However, it's worth noting that synthetic biology tends to lean towards engineering and construction metaphors when building genetic devices. As a result, the hardware analogy typically dominates, creating an electrical engineering narrative that deviates from computing—or even from computing engineering.

While mathematical Boolean functions and implementations of logic gates share many similarities, the latter are rooted in electrical engineering, a field that provides useful concepts and abstractions for biocomputing but can also be misleading. For example, focusing on engineering as the framework for biocomputing raises the issue of manipulating physical structures that evolve. Unlike hardware, living matter - or wetware - adapts and mutates in response to changing environments. This imposes a significant challenge, which will be addressed in the next section. While there are many genetic building blocks (i.e., expression systems and molecular connections) that can be arranged to build larger circuits, they lack standardization and orthogonality, which is a key feature of electronic systems. Although efforts to standardize wetware are in place^8, 38^, engineering may only be (very) useful as a *concept*. Therefore, placing the focus on the actual flow of information, from inputs to outputs, may offer a more suitable framework for manipulating living systems. For instance, genetic logic gates do not adhere to the conventional in/out standards seen in electronics, such as TTL or CMOS, and each of them can exhibit a different in/out pattern. Describing the dynamic range of each gate is the sole means of identifying compatible gates. Furthermore, in keeping with engineering topological principles, genetic circuits are typically arranged sequentially, much like their electronic counterparts. However, natural genetic networks are rich in redundancies and feedback loops – which may hold an algorithmic advantage (i.e., a better way to arrive at outputs from inputs) over intuitive forward designs^30^.

The notion that computing is only possible with silicon-based machines is a common misconception. In reality, these are just specific implementations of mathematical formalism. In fact, other implementations are possible using different forms of matter, such as living matter.

There are two types of cellular computing approaches where we can conceptually differentiate between hardware and software. The first approach involves distributed cellular computations^24, 26, 46^, which use populations of cells to implement a given function. Each cell in the population performs a specific logic function, which is then communicated to other cells as input. This allows for the function to be distributed across multiple cells, with the cells themselves acting as the hardware structures and the genetic program serving as the software. By breaking the function down into multiple parts and distributing it across cells, the computational burden can be better managed and scaled up, as cells have finite resources and energy. The second approach is reconfigurability^25^, which involves genetic devices that can be reconfigured in real-time. For example, a genetic program able to perform one of several predefined logic functions at any one time in response to a control signal^13^. This approach is closer to our understanding of software—since reconfigurable silicon-based hardware is still a challenge—and makes the devices more flexible and adaptable, as their function can be changed as needed.

In addition to the *bottom-up* approaches, involving the explicit manipulation of genetic and cellular components to create novel biological circuits, the field also explores *top-down* methods^45^. These strategies aim to understand and regulate native systems by altering input stimuli, without the need for modifying the systems themselves. They have been applied to elucidate crucial biocomputing functions, such as memory^11^, which permits the reprogramming of control strategies without necessitating adjustments to the underlying living hardware—an idea reminiscent of software utilization.

In the world of biocomputing, it's easy to get caught up in the hardware versus software debate. Because it makes a conceptual difference that matters. While both offer unique advantages, the truth is that living systems are in a league of their own. They're not hardware or software—at least how we define those—but rather a remarkable example of theoretical computations brought to life.

## 4 A tale of complexity

When it comes to cellular computing, computer science and the analysis of complex systems intersect, yet finding common ground is a significant challenge. On one hand, computer science deals with algorithmic complexity and the difficulty of solving problems. However, hardware complexity is not a major concern as the computer's physical system is predictable, and there are no unforeseen properties. On the other hand, complexity theory applied to the analysis of complex systems, such as living cells, addresses the emergent properties and nonlinearities^51^ that arise from interactions between components. Such systems are unpredictable due to a lack of complete understanding of their dynamic performance.

Biocomputing involves dealing with both fields, but their intersection remains unclear. A cell is a complex system, and designing genetic devices for computations requires consideration of the complexity of the problems and solutions that can be solved.

**Figure 3:**
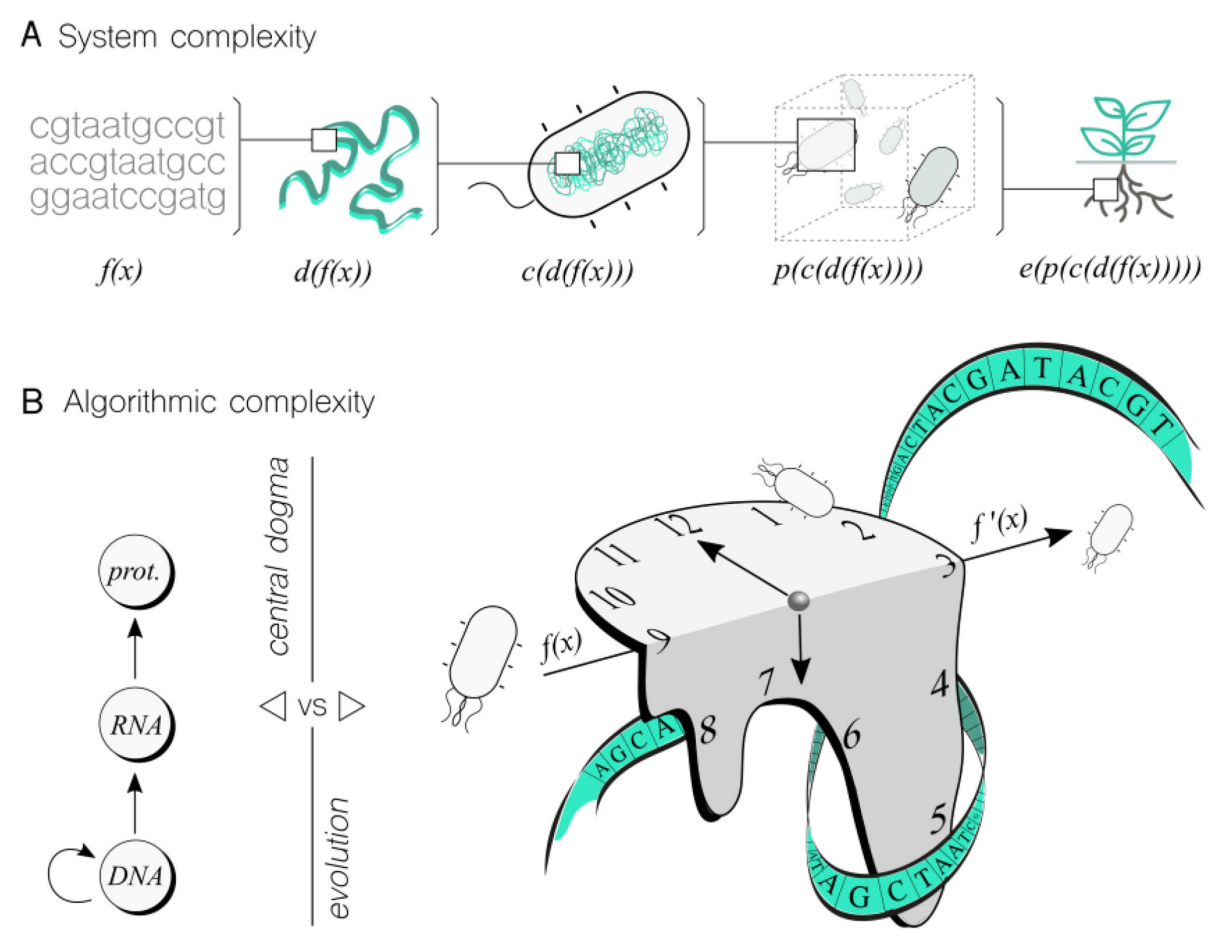
Finding common ground between systems and algorithmic complexity in complex systems and computer science. **A**. Living cells are complex systems with emergent properties resulting from interactions between constituents. To implement functions in DNA sequences, these interactions at different levels need to be considered to predict the performance of the biocomputing device. As shown in the figure, a function f(x) encoded into DNA can be modified by factors such as genomic location, host organism, population dynamics, and ecological niche, all of which add complexity to the function's performance. **B**. Algorithmic complexity is crucial to any computing endeavour and also in different living processes. While the central dogma of molecular biology (from DNA to RNA to protein) is a somewhat direct process, evolution (represented here using a Dali’s melting clock) presents much more complex information-processing capabilities. A function can evolve into a new one by adapting to ever-changing environments in an open-ended fashion, posing a significant challenge to characterising (and eventually exploiting) algorithmic complexity.

From a complex systems perspective (Figure 3A), it is important to note that the DNA sequence of a genetic program is not the only factor that affects the implementation of a specific performance. Various levels add complexity, including placing a synthetic sequence in a chromosomal location within the cell, which can introduce nonlinear changes to the original function. Additionally, the cellular chassis that carries the program, as well as the species and strain, can transform the original program. This is what we termed as *contextual dependency*^53^—a software engineering term that describes this issue. Furthermore, cells do not operate alone, and building populations with complex structures and interactions will also modify the original program. Finally, the ecological environment will introduce dynamic changes to the program's performance, which are difficult to predict in advance^49^. This latter level is of particular interest if the biocomputing device is intended to solve an environmental related problem. Despite the formidable challenge that system complexity poses for biocomputing, it should be emphasized that this challenge is not insurmountable. Living systems have evolved over millions of years to efficiently perform tasks with an astonishing level of precision while managing complexity. Of course, this does not mean that humans can learn to manipulate living matter to the same level, but we can certainly do better than we currently are.

The algorithmic complexity of biocomputing devices warrants more attention as it is a central topic in computer science. This entails addressing the question: How *difficult* is it to obtain the output within a specific biological system, given the input? Common metrics used in computing systems to tackle this question include time and space requirements. In this context, if one biological system takes longer to reach the output than another, the former is deemed more complex, at least in terms of time—assuming the output remains the same, as we are evaluating the complexity of the underlying *algorithmic* system. Letȇs consider time as a valid metric for biocomputing to illustrate the point being made here. The complexity of biological processes can be intuitively understood to have different levels (Figure 3B) in terms of the problems they can solve and the solutions they can offer. For instance, the central dogma (CD) of molecular biology describes the gene expression process (i.e., from DNA to proteins) involving transcription and translation, which can only solve problems related to DNA and the presence of cellular machinery required for expression. By conceptualizing the CD as an algorithm, we can examine the time required to complete it. In the scenario of an idealized transcriptional cascade, where the protein product of one node induces the expression of the subsequent gene in the cascade, the time will increase linearly with the cascade's length. However, this time complexity is bound to undergo alterations in response to feedback mechanisms, molecular nonlinearities, resource allocation, and other factors.

While gene expression is a relatively fast and straightforward process, evolution is much more complex and convoluted. Evolution can solve a wider range of problems, but the algorithmic solutions are not straightforward nor fast. Biological systems adapt to environmental features through evolution, which is impressive as an algorithm. Calculating the time complexity of an evolutionary process with precision, through mutation and selection steps, poses a significant challenge. However, achieving this goal would undoubtedly reveal hidden mechanistic details that could greatly benefit biocomputing efforts. While evolutionary computing draws inspiration from natural evolution, it does not fully capture its complexity and open-endedness. Therefore, harnessing the complexity of natural evolution for biocomputing purposes would be beneficial.

Many current efforts aim to overcome the inevitable effect of evolution on DNA programs, which can lead to mutation and loss of function^48^. However, the term "affected" may contribute to the problem as evolution is often viewed negatively, as a process that destroys functional programs. Instead, considering evolution as a tool can help DNA programs leverage its information processing power to increase performance beyond current limits. This way, evolution can become a valuable ally rather than an obstacle.

While achieving a comprehensive understanding of system complexity (Figure 3A) to the point of harnessing every detail seems unlikely, we can effectively abstract certain details into functional modules. This approach allows us to comprehend the input-output dynamics of modules without the need for an in-depth understanding of their internal workings. Genetic building blocks (f(x) in Figure 3A), for instance, encapsulate numerous mechanistic details, and it is not imperative to comprehend them all to utilize them in constructing fully functional genetic logic gates.

Additional examples include employing genetic gates without fully understanding their contextual dependencies^53^ (c(d(f(x))) in Figure 3A), or implementing effective multicellular distributed computations^35^ even when population dynamics are not entirely understood (p(c(d(f(x)))) in Figure 3A). Moreover, evolutionary processes can be indeed employed to design parts of a genetic circuit^56^, even though a complete description of such processes may be elusive.

In essence, to echo Donella Meadows, "we can't control [complex] systems or figure them out; but we can dance with them!" While each new insight into these systems enables more advanced biocomputations, there is still ample opportunity to leverage complexity without fully comprehending it.

## 5 Challenges and opportunities

Improving the programming of cellular computers is a challenging task that requires to strengthen the intersection of theoretical computer science and synthetic biology. For example, by formalizing new models of computation we will increase our understanding of natural living systems and enhance the scope of synthetic genetic functions. However, it's difficult to envision new models of computation based on living dynamics since we're used to the current silicon-based implementations and existing mathematical abstractions. Additionally, understanding the overlap between system and algorithmic complexity can lead to novel models of computation that are suitable for living matter. Evolution is another key challenge, as current implementations of synthetic functions tend to avoid evolutionary dynamics simply because we don’t know how to develop an engineering understanding of it^14, 29^. This becomes particularly challenging when dealing with *hard* mechanically oriented engineering disciplines as a metaphor, as is often assumed. However, the comparison becomes less challenging when contrasted with software engineering^12^. Indeed, computer science can help in this regard by providing inspiration from evolutionary computing—which already got inspiration from biology decades ago. Another interesting direction would be to move beyond the toolkit of gene regulation. Indeed, living cells are much more than that. Incorporating metabolism and other cellular processes can lead to heterotic computations^28^ that are more similar to living cells' approach to implementing key living processes.

Developing cellular computers is not only intellectually interesting but also has practical applications in various domains, from medicine^55^ to ecology^21^—even using the relatively simple cellular computers that exist today. These application domains present a unique set of classes of problems that demand living-based solutions. Moreover, classic inquiries such as the processing of morphogenetic information, which was pivotal in Turing's^20^ research, is a prominent area today that highlights the strong connections between developmental biology and information processing problems^37^. These particular challenges appear better suited for biocomputing approaches than for conventional computing methods.

There is more to come. While the limits of what human-defined cellular computers can do remain unclear, the possibilities are promising. For instance, let’s consider that microbes are ubiquitous and play essential (and beneficial) roles to systems everywhere, from our bodies to environmental ecosystems^21^. Magnificent opportunity lies ahead, which revolve around the idea of re-programming those microbes to help rebalance the system if it becomes unbalanced or collapses. Biocomputing is, without a doubt, a promising area of research to pursue.
